# Milk-Food, and the Hygienic Marketing of Same1Read before the February Meeting of the New York County Veterinary Medical Association.

**Published:** 1899-07

**Authors:** E. B. Ackerman

**Affiliations:** Brooklyn, N. Y.


### THE JOURNAL

OF

### COMPARATIVE MEDICINE AND VETERINARY ARCHIVES.

VOL. XX. *JULY, 1899.* No. 7.

### MILK-FOOD, AND THE HYGIENIC MARKETING OF SAME.[1]

BY E. B. ACKERMAN, D.V.S.,

BROOKLYN, N. Y.

DAIRY husbandry has been an industry of the people of all countries and of all times, and the production of milk as an article of human food may well be given the place of first importance. Physiologically, it contains all the proximate principles to support life readily assimilated by the human organism. The child begins its career upon milk, and much of its future depends upon the quality, both from a chemico-physiological and patho-bacteriological point of view. Aside from the child, it is largely consumed by the well and strong, not to forget the invalid, where it is second in importance to child-feeding. It has been said that the milk-supply of this country for a year amounts to 5,210,000,000 gallons, while in Greater New York City the daily supply amounts to 750,000 quarts. Our large cities spend millions of dollars annually upon their water-supply. This, of course, is a necessary expense, but equal in importance is the milk-supply from the standpoint of public health, and upon this they begrudgingly spend a few meagre thousands.

Milk, of course, both from its origin and its composition, is an animal food, and it has long been recognized even by savages that a preparatory treatment (that we call cooking) is necessary before animal foods are ready for use. This fact probably was forced upon the savages by unpleasant consequences of eating this food raw. This preparatory treatment is necessary to destroy the various harmful forms of low life which frequently

[1] Read before the February Meeting of the New York County Veterinary Medical Association.

accompany animal foods. The germ-theory of disease has shown us that many of the forms of disease most fatal to human life are due to these organisms, which are generally known as bacteria or microbes. It was not until the year 1870 that milk was generally considered capable of carrying epidemic diseases, and since that time statistics show over one hundred epidemics of typhoid fever with 6700 cases; forty-one epidemics of scarlet fever with 2400 cases, and eighteen epidemics of diphtheria with 1000 cases, to say nothing of other diseases where no records or data have been kept or no mention made as to the number of tuberculous cases established by milk.

The question of a pure milk-supply is possibly of no less interest to the medical profession than to the veterinarian, and both should work hand-in-hand for the mutual benefit of the people. While, it seems to me, our sister profession is sometimes lax in its duties in regard to the health of patients, in not being as particular about the source of milk-supply or the proper care of milk after delivery, the veterinarian, on the other hand, is interested chiefly because some of the diseases which render meat and milk unfit for human consumption are indigenous to his class of patients, namely the cow, and the animal industry represents considerable of the wealth of this country, and the care and management of this wealth largely falls upon the shoulders of the veterinarian. Take the disease tuberculosis as an example. I might say that the first prophylactic treatment begins with the veterinarian in stamping out the diseases where they start, that is, in the cow.

Now to get back to milk and to make a valuable food still more desirable, I would say that the proper place to begin is with the animal that produces it, thus begin with the dairy cow. Under this head we would have the following subdivisions, viz.:

1. The condition of the stable as to ventilation, drainage, mode of feeding and watering.
2. Health of cows. This would include all diseases, as well as the tuberculin-test for tuberculosis.
3. The feed. This includes quantity, character, water-supply, etc.
4. Care of cows. *Especially* before milking.
5. Care of utensils.
6. Health and cleanliness of attendants.

7. Care of milk. This would include the handling and shipping. Milk is an article of diet that must be kept within the reach of all at a popular price, and all improvements kept within a reasonable expenditure, the principal thing being health and cleanliness.

The diseases that may be transferred through the milk are those that the cow is subject to, and which can and do pass through the secreting organ into the milk, and those which are affected through external causes as from the organisms on the udder and skin of the cow, or on the hands or clothes of the attendants, and from the water used to wash utensils. Milk being an excellent culture-medium, and having many opportunities of being contaminated in the various handling it receives, should be removed from the stable at once to a more cleanly place, especially designated or built for the purpose of straining, aërating, and cooling. Many of the epidemics caused by milk have occurred by contamination after the milk has left the cow and before delivering to customers.

Prof. Ravenel says that ordinary milk as it reaches the consumer is usually richer in bacteria than the sewage of our great cities, and that various samples experimented with have shown anywhere from 15,000 to 4,200,000 germs per c.c. Such conditions can be greatly improved by a little carefulness and cleanliness, and belong to that part of dairy hygiene which comes under the head of purity of milk.

Now, even with the greatest of care, many germs may still be present, and to destroy these and make the milk marketable the remedy is simple. The germs of all the above diseases are absolutely destroyed by the application of a moderate heat. This process of heating applied to milk is known as "Pasteurization," or, if carried still further, "sterilization." Therefore, the remedy is, use only Pasteurized milk. It is not necessary for me to go into detail of the process of Pasteurization, for I believe you all understand it. It is subjecting milk, an animal food, to the preparatory treatment which other animal foods receive. But it may be asked, "If the remedy is so simple, why is this process not always carried out in the kitchen." Failure to appreciate the great danger of uncooked milk is doubtless one reason; the trouble and annoyance connected with the process another; an unpleasant taste and an alteration of the appearance of the milk, through the separation of stringy material, another.

The above disadvantages are avoided, the absolute safety of the milk is assured, and many other advantages are obtained by the use of the Walker process of bottling and distributing milk. This process consists in distributing Pasteurized milk in glass siphons, the siphon containing above the milk a body of insoluble gas, usually air sufficient to expel the milk. The advantages of this process may be enumerated as follows, viz.:

1. Absolute safety. The milk is Pasteurized, therefore free from disease germs. It is contained in a sealed package absolutely secure against the introduction of germs from the air.

2. You can get milk out of this siphon, but dirt, dust, or air positively cannot get in.

3. Increased keeping qualities. The spoiling of milk is due to the micro-organisms it contains. If you destroy these the milk will keep, provided it is not infected with organisms from without. Ordinary Pasteurized milk is readily infected from without, as in transferring it from the Pasteurizing vessels to the bottles, hence it does not keep much longer than ordinary milk. Milk put up by the Walker process cannot be infected from without, hence it will keep.

4. Possibility of using a part of the contents without contaminating the remainder. The instant an ordinary bottle of Pasteurized milk is opened the effect of the Pasteurizing is gone, for the contents having been exposed to the air become infected, and the condition of the milk is the same as before Pasteurization. From a siphon of milk prepared by the Walker process you may draw as much or as little as you please without affecting the contents remaining.

5. Impossibility of skimming. A siphon of Walker process milk cannot be skimmed or adulterated, either wilfully or accidentally.

Sir William Broadbent, in his address before the Society for the Prevention of Tuberculosis, says that English statistics show that nearly 60,000 deaths are every year recorded as due to tuberculosis in England and Wales, and that even this is an improvement over fifty years ago of 50 per cent. One form of disease only shows no decrease, and that is tabes mesenterica, the disease of the bowels in children traceable to tubercles conveyed by milk, and this has increased and is increasing. The one great cause of this disease is cows which have the disease, which sooner or later finds its way in the milk, as do all other diseases.

A couple of months ago, at great Smithfields, one of the best breeders of cattle, fancy stock, and dairy cows, when spoken to on the subject of tuberculosis and the dangers of milk, acknowledged the truth of it, and replied that the cure is simple. To sterilize the milk was cheap and simple, and would benefit both customer and producer, for the former would not only get pure, healthy milk, but would get it cheaper, as sterilized milk would be put up in bottles, could be kept for a long time, and could be dealt with in much the same fashion as aërated waters are. This is a fact, and this assertion is made since the Walker process patent was allowed. Society and our government have recognized the fact that it is impracticable to stamp out tuberculosis in cattle by the immediate slaughter of all diseased animals, but the public will insist that it has a right to a milk-supply absolutely free from tuberculosis and other infectious and contagious diseases, and that will not scatter death among the children of all classes of the community. Now properly conducted dairies and milk cared for by this process will reduce tabes mesenterica in children, will act well with the sick, and help keep the well from getting sick.

Prof. Stohman, of Leipsic, Germany, in Muspratt's *Chemistry*, vol. v, page 1690, says: "If it were possible to keep sterilized milk permanently free from bacteria, it would be desirable to subject all milk to this process immediately after milking, and to bring no milk into commerce except in the sterilized state. Unfortunately only a process of sterilization which is carried out in the vessels from which the milk is to be used assures a guarantee of permanent freedom from bacteria. That such a process cannot be carried out on a large scale is evident. All other processes in which the milk, after heating, comes in contact with the air afford no security that milk which has once been made germ-free will remain so. Under ordinary conditions, therefore, the sterilization of milk of commerce is of little use, as milk so treated between the time of sterilization and use has many opportunities to become infected with the most varied kinds of germs. If the cooling takes place in open coolers all sorts of germs may be carried to the milk by the air. There is the further danger of infecting the milk from improperly cleansed vessels. The milk may in the storeroom of the dealer become infected with the germs of disease. If, with the belief that germs are absent, such milk be used, it may have an injurious effect on the health, and under certain circumstances may be more injurious than if it had not been sterilized. Sterilization, whether by simply heating or boiling, or in suitable apparatus, should always be carried out at the place where the milk is to be used and a short time before use, and all processes which do not afford a guarantee of permanent freedom from bacteria should be entirely excluded."

I claim that the Walker process overcomes the only two objections Prof. Stohman offers, viz.: 1st. It does not allow of contamination after Pasteurization. 2. It is practical on a large scale—and as he says if that were practical it is the only way milk should be used.

With this process milk will keep good for two or three weeks, hence no waste. Pasteurizing it under pressure keeps the normal emulsion, and the cream does not rise of any account. It makes an improved milk to the taste, giving it more body, characteristic of cream.

I had the good luck to be present last year at a public meeting of the Philadelphia Department of Health, considering the question which was better, bottle-milk or bulk-milk in cans. The arguments were good on both sides, but the bottled milk came out victorious, having everything in its favor, and this process is as far ahead of ordinary bottled milk as bottled milk is above that in cans opened in the street every block or so.

The Abbott Dairy, of Pennsylvania, and the Walker-Gordon Dairies, of New York and Philadelphia, put up sterilized or Pasteurized milk in sealed bottles, charging a higher price for it, but only sell in pints, so you can use most of it at time of opening, else if kept it is no better than ordinary milk, and for infant-feeding the Walker-Gordon people put up Pasteurized milk, or Pasteurized modified milk, in glass tubes, each tube being a meal. Thus, if you feed the baby eight times in a day you buy eight tubes. With the Walker process you have all the tubes in one, and what you do not use to-day can go for to-morrow.

---

THE Livestock Sanitary Board of Pennsylvania will again have at its command during the ensuing year the sum of $40,000, to continue the excellent work it has been conducting in the interest of livestock owners and the consumers of animal food-products.

